# Supplementary material for: Genomic characterization of Staphylococcus aureus isolates causing osteoarticular infections in otherwise healthy children
Source: PLoS One. 2022 Aug 29;17(8):e0272425. doi: 10.1371/journal.pone.0272425 (PMC9423648; doi:10.1371/journal.pone.0272425)
Supplement: S1 Table — Assemblies were generated with short read data in Unicycler. (DOCX) [file pone.0272425.s001.docx]

**Supplementary Table** Summary statistics for *S. aureus* genome assemblies. Assemblies were generated with short read data in Unicycler.

| Isolate | Total Length | N50 | Node Count | Total sequencing Reads |
| --- | --- | --- | --- | --- |
| BJ01 | 2,901,610 bp | 345,300 bp | 78 | 2,493,902 |
| BJ02 | 2,715,651 bp | 324,653 bp | 44 | 2,573,422 |
| BJ04 | 2,883,664 bp | 141,830 bp | 93 | 2,681,288 |
| BJ05 | 2,865,130 bp | 150,065 bp | 100 | 2,749,802 |
| BJ08 | 2,796,476 bp | 684,080 bp | 70 | 3,602,990 |
| BJ09 | 2,781,240 bp | 842,798 bp | 67 | 2,833,918 |
| BJ11 | 2,874,312 bp | 493,515 bp | 67 | 1,580,456 |
| BJ12 | 2,858,770 bp | 381,724 bp | 62 | 2,730,932 |
| BJ14 | 2,838,579 bp | 150,667 bp | 91 | 3,374,678 |
| BJ16 | 2,836,748 bp | 150,664 bp | 108 | 2,628,512 |
| BJ17 | 2,742,652 bp | 324,699 bp | 49 | 2,993,488 |
| BJ18 | 2,824,929 bp | 145,010 bp | 89 | 3,035,276 |
| BJ20 | 2,859,966 bp | 379,768 bp | 73 | 2,763,582 |
| BJ22 | 2,779,014 bp | 511,094 bp | 84 | 2,970,612 |
| BJ23 | 2,677,006 bp | 410,206 bp | 65 | 3,289,726 |
| BJ26 | 2,815,461 bp | 114,527 bp | 83 | 4,828,216 |
| BJ27 | 2,716,312 bp | 122,975 bp | 108 | 3,037,438 |
| BJ30 | 2,696,579 bp | 127,949 bp | 100 | 2,847,488 |
| BJ31 | 2,799,975 bp | 150,665 bp | 76 | 2,986,212 |
| HC01 | 2,776,425 bp | 193,291 bp | 69 | 2,125,306 |
| HC02 | 2,760,837 bp | 285,743 bp | 58 | 4,037,150 |
| HC03 | 2,820,835 bp | 104,463 bp | 103 | 2,796,428 |
| HC04 | 2,820,879 bp | 128,014 bp | 102 | 2,702,478 |
| HC05 | 2,877,095 bp | 134,327 bp | 116 | 3,598,540 |
| HC06 | 2,880,941 bp | 154,342 bp | 74 | 3,488,542 |
| HC07 | 2,808,650 bp | 157,169 bp | 68 | 2,603,822 |
| HC08 | 2,823,380 bp | 621,812 bp | 53 | 2,887,710 |
| HC09 | 2,803,283 bp | 149,999 bp | 87 | 3,237,888 |
| HC10 | 2,715,641 bp | 243,762 bp | 88 | 2,884,042 |
| HC11 | 2,716,677 bp | 118,887 bp | 79 | 2,543,440 |
| HC12 | 3,392,452 bp | 105,910 bp | 125 | 2,331,754 |
| SSTI01 | 2,871,429 bp | 894,766 bp | 63 | 2,580,842 |
| SSTI02 | 2,846,042 bp | 345,301 bp | 67 | 1,969,730 |
| SSTI03 | 2,831,934 bp | 206,477 bp | 57 | 2,775,570 |
| SSTI04 | 2,834,658 bp | 681,695 bp | 72 | 2,803,116 |
| SSTI05 | 2,845,265 bp | 590,828 bp | 73 | 3,392,124 |
| SSTI06 | 2,912,114 bp | 345,300 bp | 80 | 3,087,318 |
| SSTI07 | 2,803,283 bp | 149,999 bp | 87 | 3,189,308 |
| SSTI08 | 2,802,181 bp | 150,668 bp | 85 | 2,726,460 |
| SSTI09 | 2,808,063 bp | 134,927 bp | 90 | 3,306,610 |
| SSTI10 | 2,851,911 bp | 345,300 bp | 83 | 2,358,188 |
| SSTI11 | 2,711,189 bp | 314,039 bp | 77 | 4,099,420 |
| SSTI12 | 2,792,046 bp | 195,369 bp | 75 | 2,994,698 |
| SSTI13 | 2,780,063 bp | 653,640 bp | 51 | 3,644,138 |
| SSTI14 | 2,846,853 bp | 141,745 bp | 102 | 1,860,656 |
| SSTI15 | 2,825,361 bp | 867,024 bp | 61 | 3,059,198 |
| SSTI16 | 2,897,283 bp | 345,301 bp | 80 | 4,805,946 |
