# Supplementary material for: Genomic characterization of Staphylococcus aureus isolates causing osteoarticular infections in otherwise healthy children
Source: PLoS One. 2022 Aug 29;17(8):e0272425. doi: 10.1371/journal.pone.0272425 (PMC9423648; doi:10.1371/journal.pone.0272425)
Supplement: Attachment — Submitted filename: Dehority PLoS ONE review ver2.docx [file pone.0272425.s002.docx]

The manuscript by Dehority et al describes genome scale studies aimed at determining whether there are specific genetic determinants encoded by *Staphylococcus aureus* that can be correlated with the capacity to cause invasive osteoarticular infections in children. *S. aureus* is a unique bacterial pathogen in its capacity to cause infection in essentially any human tissue, and it is very clear that it is the leading cause of bone and joint infection. For these reasons, there has been a great deal of interest in assessing the possibility that correlations exist between the presence of specific virulence factors and the capacity to cause specific forms of infection. Many studies have been published that attempted to address this issue, and given the predominance of *S. aureus* many of these have focused on bone and joint infections. None of these have reached definitive conclusions about the presence or absence of specific genes, and this manuscript is no exception. Thus, it could be argued that this manuscript adds little to the existing literature, particularly given the relatively small cohort of patients/isolates examined. However, in my view, the study was very well done and included appropriate exclusion criteria to identify isolates associated with invasive osteoarticular infection without obvious risk factors (n = 19) and controls in the form of both colonization isolates (n = 12) and isolates from “superficial” skin and soft tissue infections (n = 16). The manuscript is also very well written, and the authors appropriately note the limitation of their conclusions. The results indicated a significant correlation between the presence of immune evasion genes in isolates from osteoarticular infection vs. colonization controls, but this distinction was not present between isolates from osteoarticular vs. skin infection. There was no identifiable correlation with specific clonal lineages, although ST8 and ST30 did appear to be predominant among the isolates included in this study irrespective of infection type. The authors did identify mutations in 14 genes involved in biofilm formation, but as with the immune evasion genes the distinction was between isolates from osteoarticular infection and the colonization controls but not between osteoarticular isolates and isolates from skin infection. Additionally, these “mutations” appear to have been defined by comparison to the NCTC8325 genome, and there is no description of how they were defined as mutations beyond that. This assumes that NCTC8325, which is NOT a contemporary clinical isolate, can be defined as a wild-type strain, and there is considerable data in the literature to suggest that this is not the case. In this regard, skin infections are also considered biofilm associated infections, so it would have been of interest if the authors had examined these correlations at a phenotypic level (i.e. did the colonization strains and infection strains have a significant difference in their capacity to form a biofilm?). Similarly, the primary conclusion from the manuscript is that “genetic drivers of *S. aureus* osteoarticular infections may be primarily regulated by factors other than genomic content (e.g. at the transcriptional or translational level)”, and I believe the manuscript would be significantly enhanced if this possibility had been examined. Indeed, the authors even note a previous manuscript that found differences in the expression of *hla*, which along with protein A is generally considered a strong indicator differences in regulatory circuits in *S. aureus* and, like biofilm formation, is an easy phenotype to assess.
