# Supplementary material for: Genomic characterization of Staphylococcus aureus isolates causing osteoarticular infections in otherwise healthy children
Source: PLoS One. 2022 Aug 29;17(8):e0272425. doi: 10.1371/journal.pone.0272425 (PMC9423648; doi:10.1371/journal.pone.0272425)
Supplement: Attachment — Submitted filename: 6-12-22 Response to Reviewers PLoS ONE.docx [file pone.0272425.s003.docx]

**Response to Reviewers**
Note that we have only presented the questions and specific requests from the overall comments from the reviewers in an effort to simplify the response letter.

**Reviewer #1**

1.) A more meaningful, and somewhat objective, way to explore clinical differences between S. aureus isolates that led to invasive infection of bone (and/or joint) is the calculation of ‘Severity of Illness’ from readily available clinical and laboratory parameters which are identified during the first few days of hospitalization. It would be very interesting to see how clinical severity of illness maps out according to the phylogenetic relationship of the OAI pathogens in this study.

**Response: An excellent suggestion, and we were able to do that. We used the scoring system proposed by Athey, et al in 2019 (Athey, et al. J Pediatr Orthop. 2019;39:90-97. PMID 27741035), which was a slight modification of a prior scoring system (Copley et al. Pediatr Infect Dis J. 2014;33:35-41. PMID 24352188) to categorize all subjects with acute osteomyelitis in our study. Severity scores ranged from 1 to 5. These scores were incorporated into the phylogeny tree in Figure 1, with updates to the figure legend. Additional methods verbiage was added to lines 151-157 as well as descriptions in the results section (lines 247-248) and the legend to Figure 1 regarding these additions. Note that we also re-generated the phylogeny to get the bootstrap support values for Figure 1. The regenerated tree in Figure 2 has some minor cosmetic changes relative to the original submission (e.g. rotated nodes). We also recreated Figure 2 so that tree structures are identical in both figures.**

2.) Staph aureus does not have a lot of major genetic recombinations or mutations. It most likely evolves through single nucleotide polymorphisms and lateral transfer of small amounts of genetic material through plasmids. Phylogenetic analysis is therefore challenging as the analysis performed over 3 ½ years of enrollment for a small number of isolates is only a mathematical estimation of genetic distance between isolates and does not imply phylogeny or descent from related pathogens. That should be acknowledged in the discussion.

**Response: We have added denotations indicating nodes with ultrafast bootstrap support of at least 95% in Figure 1, which provides information on uncertainty in the presented phylogenetic relationships, and referenced this in the legend to Figure 1. We have also added verbiage to this effect in the discussion section, lines 428-432, as well as a new reference to this effect (#50).**

**Reviewer #2**

1.) Additionally, these “mutations” appear to have been defined by comparison to the NCTC8325 genome, and there is no description of how they were defined as mutations beyond that. This assumes that NCTC8325, which is NOT a contemporary clinical isolate, can be defined as a wild-type strain, and there is considerable data in the literature to suggest that this is not the case. In this regard, skin infections are also considered biofilm associated infections, so it would have been of interest if the authors had examined these correlations at a phenotypic level (i.e. did the colonization strains and infection strains have a significant difference in their capacity to form a biofilm?).

**Response: An excellent point. This is a limitation of the study, and, as you correctly note, one that is discussed in the literature.   However, given the extensive annotation of the NCTC8325 strain, the fact that it is still in use as a reference genome, and that it is familiar to investigators we think it still has validity as a comparator. Further, our use of internal comparisons with multiple clinical controls and the phenotypic investigation/validation of the noted biofilm mutations (suggested by you and discussed below), would suggest that our findings have merit.  We would also note that the new phenotypic biofilm assessments, as well as the extensive phylogenetic analyses (including the newly added stratification by severity of illness) coupled with the use of our multiple internal control groups are all novel approaches in this subject population.  We have added verbiage to this effect to the discussion section, in lines 461-466**.

**To attempt to address this issue further, and to incorporate your biofilm suggestions, we performed analyses to assess capacity to form biofilm with the isolates. Interestingly, no difference existed in the ability of the infection-associated isolates to form biofilm, though isolates taken from healthy controls were significantly more robust in this regard. New verbiage addressing the biofilm assays has been added to lines 37-38 and 44-45 in the abstract, in the methods section in lines 158-173 (describing the experimental approach) and lines 189-195 (describing the statistical approach to analyzing results), in the results section (lines 347-359), in the discussion section (lines 412-414 and lines 446-455) and in Figure 4. We have also added Seth Daly, Kathleen Triplett and Pamela Hall (and acknowledged the NIH funding of Dr. Hall) to the manuscript, as they assisted with the biofilm assays.**

2.) Similarly, the primary conclusion from the manuscript is that “genetic drivers of S. aureus osteoarticular infections may be primarily regulated by factors other than genomic content (e.g. at the transcriptional or translational level)”, and I believe the manuscript would be significantly enhanced if this possibility had been examined.

**Response:** **We agree with this viewpoint. We discussed this option extensively as the study was being planned, but we felt we did not have the resources or finances to pursue transcriptional or translational analyses at the time, and as a result, focused on genomic analyses. Following this study, however, we would like to incorporate these into future work.**
